# Identifying and validating subtypes of Parkinson's disease based on multimodal MRI data *via* hierarchical clustering analysis

**DOI:** 10.3389/fnhum.2022.919081

**Published:** 2022-07-29

**Authors:** Kaiqiang Cao, Huize Pang, Hongmei Yu, Yingmei Li, Miaoran Guo, Yu Liu, Guoguang Fan

**Affiliations:** ^1^Department of Radiology, The First Affiliated Hospital of China Medical University, Shenyang, China; ^2^Department of Neurology, The First Affiliated Hospital of China Medical University, Shenyang, China

**Keywords:** Parkinson's disease subtypes, cluster analysis, magnetic resonance imaging, amplitude of low-frequency fluctuations (ALFF), gray matter volume (GMV)

## Abstract

**Objective:**

We wished to explore Parkinson's disease (PD) subtypes by clustering analysis based on the multimodal magnetic resonance imaging (MRI) indices amplitude of low-frequency fluctuation (ALFF) and gray matter volume (GMV). Then, we analyzed the differences between PD subtypes.

**Methods:**

Eighty-six PD patients and 44 healthy controls (HCs) were recruited. We extracted ALFF and GMV according to the Anatomical Automatic Labeling (AAL) partition using Data Processing and Analysis for Brain Imaging (DPABI) software. The Ward linkage method was used for hierarchical clustering analysis. DPABI was employed to compare differences in ALFF and GMV between groups.

**Results:**

Two subtypes of PD were identified. The “diffuse malignant subtype” was characterized by reduced ALFF in the visual-related cortex and extensive reduction of GMV with severe impairment in motor function and cognitive function. The “mild subtype” was characterized by increased ALFF in the frontal lobe, temporal lobe, and sensorimotor cortex, and a slight decrease in GMV with mild impairment of motor function and cognitive function.

**Conclusion:**

Hierarchical clustering analysis based on multimodal MRI indices could be employed to identify two PD subtypes. These two PD subtypes showed different neurodegenerative patterns upon imaging.

## Introduction

Parkinson's disease (PD) was first described in an article written by British physician James Parkinson in 1817. PD is a neurodegenerative disease with a high incidence worldwide. PD pathogenesis is not clear and therapy to prevent PD progression is not available. PD is considered to be highly heterogeneous, with motor symptoms and a wide range of non-motor symptoms (Chaudhuri et al., 2006; Kalia and Lang, 2015). The heterogeneity of PD suggests that there may be different subtypes of the disease (Foltynie et al., 2002). Patients with the same subtype of PD are likely to have identical pathophysiological characteristics. Identification of different subtypes contributes to pathophysiological research but also has an impact on understanding of disease progression, prognosis, and “personalized” treatment.

Jankovic and colleagues were the first to postulate that PD can be divided into two subtypes: postural instability and gait difficulty-dominant (PIGD) and tremor-dominant (TD) (Jankovic et al., 1990). This is the most widely used subtype classification in the clinic. However, a significant limitation of this approach is assigning patients to a specific subtype based solely on motor symptoms without considering the complexity of the clinical symptoms of PD. Recent research has suggested that TD and PIGD are unstable and interchangeable over time (Simuni et al., 2016; Erro et al., 2019).

Several studies have explored the different subtypes of PD through data-driven unsupervised cluster analysis (Erro et al., 2016; Fereshtehnejad et al., 2017; Belvisi et al., 2021). However, most of the cluster analysis carried out so far is characterized by a clinical scale score, which lacks the support of objective biomarkers or imaging data, and is subjective (Hendricks and Khasawneh, 2021). Undertaking cluster analysis using more objective disease characteristics combining motor symptoms and non-motor symptoms is a rational approach.

Neuroimaging provides numerous potential biomarkers for neurodegenerative diseases. Resting-state functional magnetic resonance imaging (Rs-fMRI) has been used widely. Measuring the blood oxygen level-dependent (BOLD) signal amplitude of low-frequency fluctuation (ALFF) can reflect local spontaneous neuron activity effectively (Zang et al., 2007). ALFF shows high retest reliability (Zuo and Xing, 2014), which ensures its reliability as an indicator to detect individual regional functional differences (Zuo et al., 2019).

Voxel-based morphometry (VBM) is an efficient method for analyses of MR images of brain structure. VBM overcomes the influence of individual differences and is highly sensitive to shrinkage of gray-matter volume (GMV) (Pereira et al., 2012). VBM has been used widely to quantify GMV changes in PD patients (Xu et al., 2016, 2020). Studies have demonstrated that the changes in patterns of ALFF and GMV are significantly different in different PD patients (Chen et al., 2015; Ma et al., 2018). However, studies combining ALFF with GMV to explore PD subtypes have not been done.

We aimed to identify different PD subtypes based on hierarchical clustering analysis of ALFF and GMV. We wished to further analyze the change in patterns of ALFF and GMV among different subtypes. In this way, we aimed to help provide more accurate personalized treatment. We hypothesized that cluster analysis based on multimodal indicators could accurately identify PD subtypes characterized by different neurodegenerative patterns. Moreover, different PD subtypes have unique neurophysiological bases.

## Materials and methods

### Subjects

Eighty-six PD patients admitted to the Department of Neurology of the First Affiliated Hospital of China Medical University from June 2019 to September 2021 were enrolled. All PD patients met the Brain Bank Diagnostic Criteria of Parkinson's Disease Association UK (Hughes et al., 1992). We also recruited 44 healthy controls (HCs) matched for age, sex, and education level. For patients taking anti-PD drugs, MRI and clinical examination were undertaken ≥12 h after drug withdrawal. The clinical measurements were Unified Parkinson's Disease Rating Scale Part III (UPDRSIII), Hoehn–Yahr (H–Y) scale, Montreal Cognitive Assessment (MoCA), Mini-Mental State Examination (MMSE), and Hamilton Depression Scale (HAMD). The exclusion criteria were patients: (1) with a history of brain tumors, cerebrovascular diseases, or other mental disorders; (2) with a history of drug abuse; (3) suffering from hypertension, diabetes mellitus, or a respiratory disease; (4) who were not right-handed; (5) with contraindications to MRI examination. The study protocol was approved by the Ethics Review Committee of China Medical University (Shenyang, China). All participants provided written informed consent.

### Acquisition of MRI data

Imaging data were obtained on a 3.0-T MRI scanner (Magnetom Verio; Siemens, Erlangen, Germany) with a 32-channel head coil. Participants were instructed to close their eyes, relax, and remain conscious. Functional images were obtained using an echo-planar imaging sequence: echo time (TE) = 30 ms; repetition time (TR) = 2500 ms; field of view (FOV) = 224 mm × 224 mm; matrix = 64 × 64; voxel size = 3.5 mm × 3.5 mm × 3.5 mm; slice numbe*r* = 43; slice gap/thickness = 0/3.5 mm; flip angle = 90°; total volume = 240.

High-resolution T1-weighted images were obtained using a magnetization-prepared rapid gradient echo (MPRAGE) sequence (TR = 5000 ms; TE = 2960 ms; FOV = 256 mm × 256 mm; matrix = 256 × 256; voxel size =1.0 mm × 1.0 mm × 1.0 mm; slice numbe*r* = 176; slice gap/thickness = 0/1 mm; flip angle = 12°).

### Data preprocessing

Data Processing Assistant for Rs-fMRI (DPARSF; http://rfmri.org/DPARSF) and SPM12 (http://www.fil.ion.ucl.ac.uk/spm) were used to preprocess fMRI data according to standardized processing procedures (Yan et al., 2016). There were six major steps. First, the first 10 volumes of functional images were discarded. Second, slice timing and correction of head movements were undertaken by excluding people with head movement >2.5 mm or rotation >2.5°. Third, spatial normalization of structure and registration of functional images were done through DARTEL. Fourth, we carried out linear regression of disturbance covariables (head parameters, white-matter signals, cerebrospinal-fluid signals). Fifth, a 6-mm full-width at half maximum (FWHM) Gaussian kernel was employed for spatial smoothing. Sixth, we removed the linear trend.

ALFF was the root mean square of the BOLD-signal power spectrum between 0.01 Hz and 0.08 Hz for each voxel. Next, we divided the global mean value to reduce the impact of variability among participants. Anatomical Automatic Labeling (AAL) is provided by the Montreal Neurological Institute (Montreal, Canada) and divides the human brain into 116 regions. It is used widely in neuroimaging research, and has good reliability. We used a cutoff of 2 to extract the ALFF values of 116 brain regions.

For high-resolution T1-weighted images, we followed standard procedures for VBM analysis using the SPM12-based Computational Anatomy Toolbox (CAT12; http://dbm.neuro.unijena.de/cat/). The major steps are spatial normalization, correction of bias fields, segmentation into gray matter, white matter, and cerebrospinal fluid and, finally, gray-matter images are smoothed with an 8-mm FWHM Gaussian kernel. In addition, we used the “Modulation” feature to compensate for the effect of spatial normalization. We defined total GMV as the summation of the GMVs of all voxels. We used the AAL atlas to segment GMV maps and extract values (Jiang et al., 2021).

### Cluster analysis

**Figure 1:**
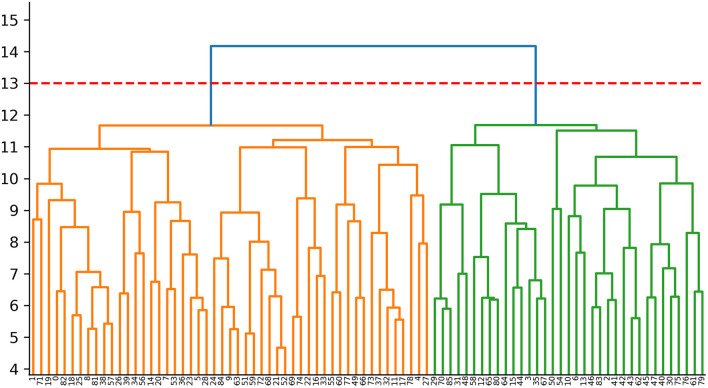
Dendrogram of the cluster analysis. Cluster 1:The orange area on the left (*n* = 51,59.3%); Cluster 2:The green area on the right (*n* = 35, 40.7%).

We extracted ALFF and GMV from 116 brain regions as clustering features for each PD patient, and conducted dimensionless processing through standardization. To reduce the data dimension and improve the clustering performance of the model, we used principal components analysis (PCA) for dimensionality reduction, and took eigenvalue >1 as the selection criterion for principal components. In hierarchical clustering analysis, each patient was first treated as a separate “cluster” and then merged gradually with other patients into a new cluster. We used the Ward linking method to merge clusters at each step while minimizing the sum of error squares from the cluster mean (Uribe et al., 2016, 2018; Inguanzo et al., 2021). The results of cluster analysis were shown as a dendrogram (Figure 1). We used the Calinski–Harabasz (C–H) Index to assess the optimal number of clusters. This was determined by between-cluster and within-cluster variance, and the larger the value, the better was the cluster solution. We implemented the operations stated above using the “sklearn” package on the Anaconda3 platform (www.anaconda.com).

### Statistical analyses

Demographic and clinical data were analyzed using SPSS 26.0 (IBM, Armonk, NY, USA). The normality of data distribution was assessed using the Kolmogorov–Smirnov test. The age, education level, as well as scores for MoCA, MMSE, and HAMD among the three groups were analyzed by the Kruskal–Wallis H test. Sex was analyzed by the chi-square test. The Mann–Whitney *U*-test was employed to test for disease duration, UPDRSIII score, and H–Y score between the two clusters. *P* < 0.05 was considered significant.

Data Processing and Analysis for Brain Imaging (DPABI) was used for the statistical analyses of imaging data. We undertook one-way analysis of covariance (ANCOVA) with age and sex as covariates to explore ALFF changes among cluster 1, cluster 2, and HCs (Gaussian random field (GRF) correction; voxel size: *p* < 0.005; cluster size: *p* < 0.05). Also, we used the two-sample *t*-test to assess the difference in ALFF between groups, with age and sex as covariates (within a mask having significant differences in the ANCOVA). We used ANCOVA with age, sex, and total intracranial volume as covariates to explore GMV differences among the three groups (GRF correction; voxel size: *p* < 0.005; cluster size: *p* < 0.05). The method of comparison between groups was consistent with ALFF. Covariate and multiple methods for comparison correction were consistent with ANCOVA. Considering that our data did not conform to a normal distribution, Spearman correlation analysis was undertaken to investigate the correlation of ALFF and GMV with clinical characteristics. *P* < 0.05 was considered significant.

## Results

### PD subtypes based on cluster analysis and clinical data

The study cohort comprised 86 PD patients and 44 HCs. We ranked the independent principal components generated during reduction of PCA dimensionality in the order of decreasing variance. Then, we selected the first 34 principal components with eigenvalue >1, and 89.45% of the effective information was retained (Supplementary Table 1; Supplementary Figure 1). PD was divided into two subtypes based on hierarchical clustering analysis of multimodal MRI indices. The neurodegenerative pattern of patients in cluster 1 (*n* = 51, 59.3%) was characterized by reduced ALFF in the visual-related cortex and extensive reduction in GMV. Patients in cluster 2 (*n* = 35, 40.7%) were characterized by increased ALFF in frontal, temporal, and sensorimotor cortices and mild reduction in GMV. For hierarchical clustering, we chose the two-cluster solution because it had the highest CH value (3.13). The CH value of the three-cluster, four-cluster, and five-cluster solutions was 2.34, 2.31, and 2.29.

Statistical analyses revealed no significant differences in age, sex, or years of education between the two PD subtypes and HC group. Compared with HCs, patients with both subtypes had lower scores for MMSE and MoCA, and a higher HAMD score. Moreover, the differences in scores for MMSE, MoCA, and UPDRSIII between the two subtypes were significant. Compared with patients in cluster 2, patients in cluster 1 had lower scores for MMSE and MoCA and a higher UPDRSIII score. Significant differences were not observed for disease duration, H–Y Scale score, or HAMD score between the two subtypes (Table 1). For an identical disease duration, cluster-1 patients showed more severe impairment in motor function and cognitive function than cluster-2 patients. In consideration of other studies (Fereshtehnejad et al., 2015, 2017; Belvisi et al., 2021), we referred to cluster 1 as the “diffuse malignant subtype” and cluster 2 as the “mild subtype”.

**Table 1:** Demographic and clinical characteristics of PD subtypes.

Domain	**Cluster1**	**Cluster2**	**HC**	χ^2^**/Z**	***p*** **-value**
	**(*****n** =* **51)**	**(*****n** =* **35)**	**(*****n** =* **44)**		
Age (years)	61.47 ± 8.46	62.09 ± 7.97	59.77 ± 7.61	2.038	0.361
Sex (M/F)	21/30	23/12	22/22	5.016	0.081
Education (years)	10.41 ± 3.19	10.26 ± 3.45	11.82 ± 2.99	4.468	0.107
Disease duration (years)	5.03 ± 3.46	5.67 ± 3.02	—	−1.356	0.175
H-Y	2.16 ± 0.79	2.21 ± 0.68	—	−0.785	0.432
UPDRSIII	33.08 ± 16.51	25.69 ± 12.10	—	−2.307	0.021
MMSE	24.37 ± 2.15	26.40 ± 2.28	28.02 ± 2.03	49.723	0.000^*^^a^
MoCA	21.25 ± 3.43	23.66 ± 3.62	25.48 ± 2.13	37.206	0.000^*^^b^
HAMD	11.98 ± 6.97	9.86 ± 8.42	1.75 ± 3.04	60.282	0.000*

Variables are mean ± standard deviation; HC, healthy controls; H-Y, Hoehn-Yahr; UPDRS III, Unified Parkinson's disease Rating Scale part III; MMSE, Mini-Mental State Examination; MoCA, Montreal Cognitive Assessment; HAMD, Hamilton Depression Scale; p < 0.05 was considered statistically significant; 0.000 ^*^ = values < 0.001; a = Comparison between the two subtypes p < 0.001; b = Comparison between the two subtypes p = 0.004.

### ALFF analysis

**Figure 2:**
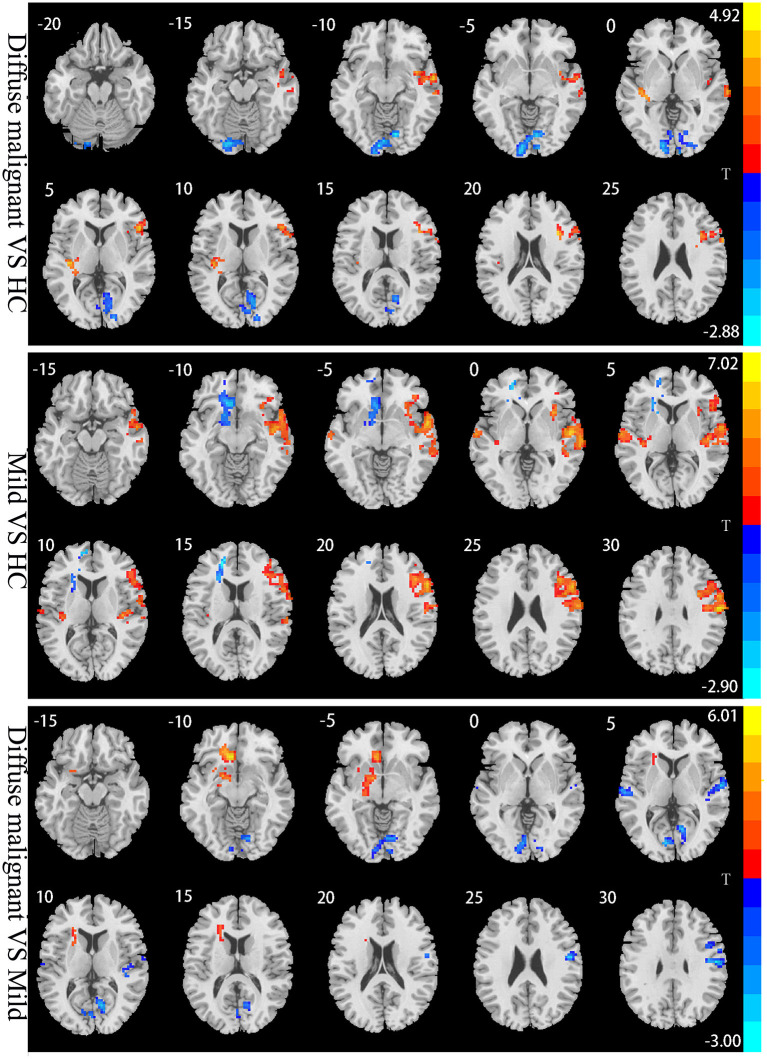
Statistical parametric map showing the significant differences in the ALFF between three groups: Diffuse malignant subtype, Mild and HC. GRF corrected (Voxel *p* < 0.005, cluster *p* < 0.05). The color bar on the right indicates the statistical t-value. Warm (cold) overlays indicate higher (lower) ALFF in PD patients.

Compared with HCs, patients with the diffuse malignant subtype had lower ALFF in the bilateral primary visual cortex, visual association cortex (bilateral lingual gyrus, right precuneus), and left cerebellum. ALFF of the right dorsolateral prefrontal cortex (DLPFC), supplementary motor area (SMA), right superior temporal gyrus, middle temporal gyrus, and insula was increased significantly. Compared with HCs, patients with the mild subtype had more extensive changes in ALFF: decreased ALFF in the left striatum, left anterior cingulate gyrus, and left medial prefrontal lobe, and significantly increased ALFF in bilateral superior temporal gyrus, middle temporal gyrus, transverse temporal gyrus, right dorsolateral prefrontal lobe, right anterior/posterior central gyrus, and right insula. Compared with the mild subtype, patients with the diffuse malignant subtype had decreased ALFF in the bilateral vision-related cortex, right anterior/posterior central gyrus, bilateral superior temporal gyrus, and right transverse temporal gyrus, and increased ALFF in the left anterior cingulate gyrus, left medial prefrontal lobe, and left striatum (Table 2; Figure 2).

**Table 2:** Brain regions exhibiting an altered ALFF among three groups.

**Brain regions (AAL)**	**Cluster size**	**MNI coordinates**			**Peak** ***t*****-value**
		***x***	***y***	***z***	
**Diffuse malignant vs. HC**					
Calcarine_L/R	113/110	15	−63	9	−2.88148
Lingual_L/R	83/80				
Cerebelum_Crus1_L	17				
Cuneus_R	12				
Frontal_Inf_Tri_R	65	39	18	18	4.91613
Frontal_Inf_Oper_R	64				
Precentral_R	14				
Temporal_Sup_R	76	60	−3	−9	4.64539
Temporal_Mid_R	65				
Insula_R	14				
**Mild vs. HC**					
Temporal_Sup_R	302	57	−12	30	7.02314
Frontal_Inf_Oper_R	170				
Frontal_Inf_Tri_R	161				
Postcentral_R	144				
Temporal_Mid_R	137				
Precentral_R	93				
Temporal_Pole_Sup_R	63				
Insula_R	24				
Heschl_R	21				
Frontal_Mid_R	19				
Putamen_L	24	−21	3	−6	−2.89661
Cingulum_Ant_L	22				
Frontal_Sup_Medial_L	17				
Caudate_L	15				
Insula_R	34	33	15	0	5.31788
Frontal_Inf_Orb_R	26				
Temporal_Sup_L	58	−60	−9	3	5.61918
Temporal_Mid_L	18				
Postcentral_R	37	39	−30	39	5.00571
Heschl_L	13	−36	−24	6	4.57343
Diffuse malignant vs. mild
Calcarine_L/R	74/68	12	−75	−12	−2.88648
Lingual_L/R	34/62				
Postcentral_R	59	60	−3	27	−2.89242
Precentral_R	26				
Precentral_R	29	42	−15	48	−3.00327
Postcentral_R	25				
Temporal_Sup_R	54	51	−18	9	−2.89128
Heschl_R	11				
Temporal_Sup_L	33	−54	−12	6	−2.89656
Cingulum_Ant_L	19	−6	27	−9	6.01187
Frontal_Sup_Orb_L	11				
Putamen_L	11	−21	21	12	4.04517
Putamen_L	10	−12	0	−6	4.68472
Pallidum_L	10				

### GMV analysis

**Figure 3:**
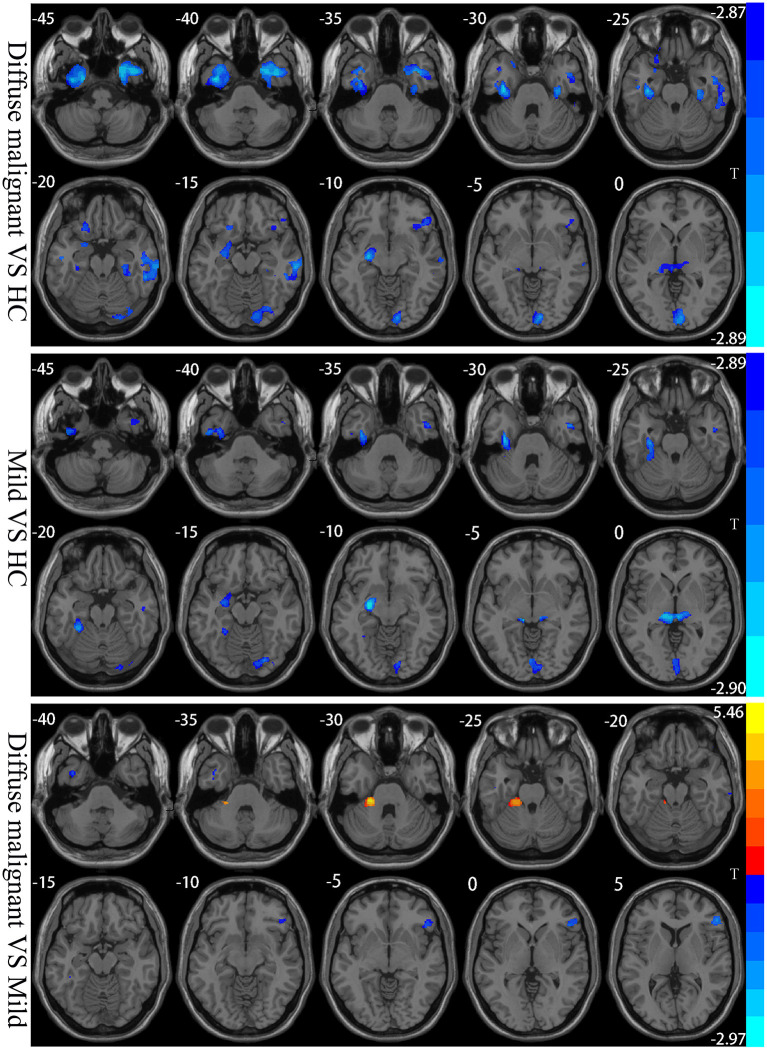
Statistical parametric map showing the significant differences in the GMV between three groups: Diffuse malignant subtype, Mild and HC. GRF corrected (Voxel *p* < 0.005, cluster *p* < 0.05). The color bar on the right shows the statistical t-value. Warm (cold) overlays show higher (lower) GMV in PD patients.

Compared with HCs, patients with the diffuse malignant subtype had reduced GMV in bilateral inferior temporal gyrus, middle temporal gyrus, inferior frontal gyrus, fusiform gyrus, parahippocampal gyrus, calcarine cortex, lingual gyrus, precuneus, thalamus, right cerebellum, left amygdala, and hippocampus. Patients with the mild subtype had reduced GMV in the bilateral calcarine cortex, cuneus, lingual gyrus, fusiform gyrus, inferior temporal gyrus, thalamus, cerebellum, right middle temporal gyrus, left hippocampus, and amygdala. Compared with the mild subtype, patients with the diffuse malignant subtype had reduced GMV in the right inferior frontal gyrus triangle, orbital region, and bilateral inferior temporal gyrus, and increased GMV in the left cerebellum (Table 3; Figure 3).

**Table 3:** Brain regions exhibiting an altered GMV among three groups.

**Brain regions (AAL)**	**Cluster size**	**MNI coordinates**			**Peak** ***t-*****value**
		***x***	***y***	***z***	
**Diffuse malignant vs. HC**					
Temporal_Inf_R	2082	60	−42	−15	−2.87966
Fusiform_R	770				
Temporal_Mid_R	601				
Temporal_Pole_Mid_R	520				
ParaHippocampal_R	187				
Fusiform_L	938	−31.5	−30	−21	−2.87905
Temporal_Inf_L	805				
Temporal_Pole_Mid_L	227				
ParaHippocampal_L	79				
Temporal_Mid_L	66				
Calcarine_L/R	563/434	1.5	−73.5	19.5	−2.87914
Cuneus_L/R	514/115				
Lingual_L/R	38/467				
Cerebelum_Crus1_R	112				
Cerebelum_6_R	91				
Fusiform_R	88				
Frontal_Inf_Orb_L	217	−21	−4.5	−7.5	−2.88584
Amygdala_L	199				
Hippocampus_L	183				
Thalamus_L/R	332/56	−15	−30	−3	−2.87827
Hippocampus_L	76				
Frontal_Inf_Orb_R	405	45	36	−13.5	−2.87847
**Mild vs. HC**					
Calcarine_L/R	543/112	4.5	−88.5	9	−2.89519
Cuneus_L/R	531/307				
Lingual_ L/R	70/312				
Fusiform_R	66				
Cerebelum_6_R	62				
Cerebelum_Crus1_R	54				
Fusiform_L	894	−34.5	−10.5	−45	−2.89446
Temporal_Inf_L	162				
Cerebelum_4_5_L	131				
Cerebelum_6_L	48				
Thalamus_L/R	514/282	−15	−34.5	10.5	−2.89847
Hippocampus_L	165	−22.5	0	−16.5	−2.89493
Amygdala_L	144				
Temporal_Inf_R	224	51	−6	−27	−2.89371
Temporal_Mid_R	79				
Diffuse malignant vs. Mild
Frontal_Inf_Tri_R	534	51	34.5	−7.5	−2.96526
Frontal_Inf_Orb_R	208				
Cerebelum_4_5_L	338	−24	−30	−31.5	5.45874
Cerebelum_3_L	37				
Temporal_Inf_L	112	−39	−3	−39	−2.968
Temporal_Inf_R	18	67.5	−24	−24	−2.96275

### Correlation analysis

**Figure 4:**
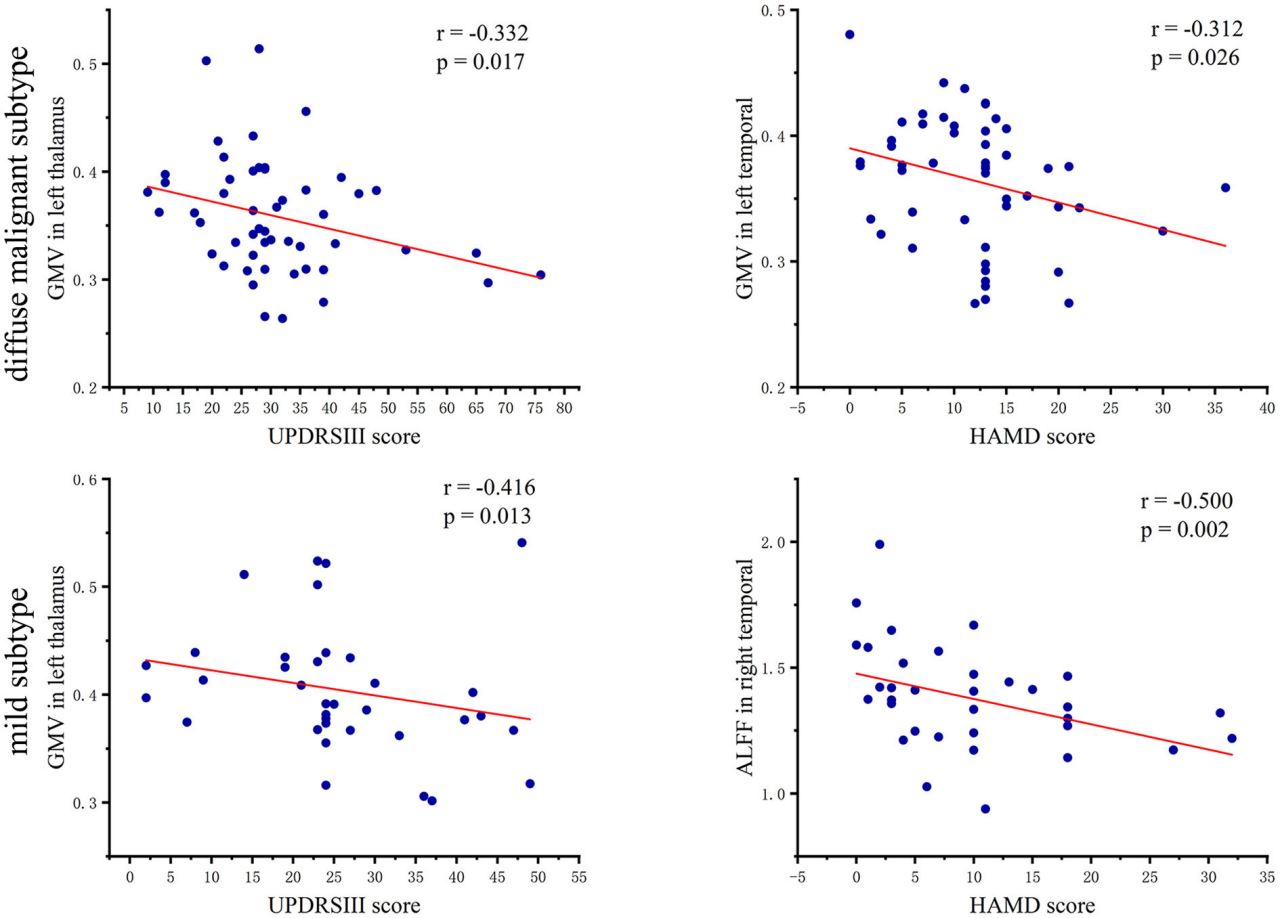
Scatter plot of correlation coefficient between PD subtypes and clinical symptoms.

In patients with the diffuse malignant subtype, the UPDRSIII score showed a significant negative correlation with GMV in the left thalamus (*r* = −0.332, *p* = 0.017), and the HAMD score had a significant negative correlation with GMV in the left temporal lobe (*r* = −0.312, *p* = 0.026). In patients with the mild subtype, the UPDRSIII score had a negative correlation with GMV in the left thalamus (*r* = −0.416, *p* = 0.013), However, the HAMD score showed a significant negative correlation with ALFF in the right temporal lobe (*r* = −0.500, *p* = 0.002). A scatter plot of the correlation coefficient is shown in Figure 4.

## Discussion

We identified two PD subtypes according to a clustering analysis of change patterns in ALFF and GMV. The diffuse malignant subtype was characterized by reduced ALFF in the visual-related cortex and extensive reduction of GMV with severe impairment in motor function and cognitive function. The mild subtype was characterized by increased ALFF in the frontal lobe, temporal lobe, and sensorimotor cortex, along with a slight decrease in GMV with mild impairment in motor function and cognitive function.

Patients with the diffuse malignant subtypes had decreased ALFF in the bilateral primary visual cortex and visual association cortex, which handle the processing and transmission of visual information. Consistent with our data, Yao and coworkers (Yao et al., 2015) found that reduced ALFF in the lingual gyrus was associated with hallucinations in PD patients. Boecker and collaborators (Boecker et al., 2007) found through positron emission tomography that the glucose metabolic rate of the vision-related cortex decreased, which helped to explain the visual impairment observed in PD patients. We also found that ALFF in the left cerebellum was reduced, which is consistent with the study results of Skidmore and colleagues (Skidmore et al., 2013) and Zhang and collaborators (Zhang et al., 2013). The cerebellum is believed to be involved in motor regulation and cognitive regulation through the cerebello-thalamo-cortical pathway, and has pathological and compensatory roles in PD. We found only a reduction in cerebellar ALFF, which may be the pathological change caused by degeneration of dopaminergic neurons. However, a cerebellar compensatory effect was not observed, which may be because the cerebellar compensatory effect is evident in the early stage of PD but weakens gradually (or even disappears) with the severity of pathological damage (Jankovic, 2005). In our study, the mean duration of PD was 5 years, which is not the early stage of PD.

Our findings further confirm that the cerebellum is involved in the pathological changes observed in PD. Compared with HCs, patients with the diffuse malignant subtype had increased ALFF in the right DLPFC, SMA, and insula, which are vital components of the task-positive network (TPN). The latter antagonizes the default mode network (DMN) and provides top–down attention direction, which plays an important part in motion control (Fox et al., 2005). Consistent with our study results, Boord and colleagues and Maidan et al. (Boord et al., 2017; Maidan et al., 2019) found increased activation of the TPN in PD patients. They pointed out that the neural processing efficiency of the TPN in PD patients was reduced, so activation of more neurons was required to maintain normal function. Besides the TPN, we also found an increase of ALFF in the right temporal lobe, which is consistent with data from other studies (Wang et al., 2020). The exact mechanism of action underlying these findings is not clear, but we speculate that the increase in the right TPN and temporal ALFF in patients with the diffuse malignant subtype reflects a compensatory function.

Compared with HCs, patients with the mild subtype had reduced ALFF in the left striatal lobe, which suggests reduced neuronal activity in this region as a critical node in the striato-thalamo-cortical pathway. The change in its functional activities has an important influence on the impairment in motor function and cognitive function of PD patients. Pathological studies have shown that α-synuclein deposition in the striatal neurons of PD patients further aggravates damage to the dopaminergic system (Agosta et al., 2014): our findings further support this conclusion. We also found that ALFF decreased in the left anterior cingulate gyrus and medial prefrontal lobe, thereby suggesting DMN injury. The DMN is a functional brain network with enhanced activity in the resting state. It is involved in episodic memory, emotional processing, self-reflection, and maintenance of consciousness awakening (Greicius et al., 2004). Several studies have shown that PD patients have damaged and reduced functional connectivity in the DMN (Greicius et al., 2004; Harrington et al., 2017), which is consistent with our research results. Compared with HCs, we found increased ALFF in the right DLPFC and insula in patients with the mild subtype, which suggested that patients with the mild subtype had compensatory functions in the TPN similar to those of patients with the diffuse malignant subtype. Therefore, we speculate that functional compensation of the TPN may be prevalent in PD patients.

We also found that patients with the mild subtype had increased ALFF in the right sensorimotor cortex, in which the right anterior central gyrus have an important role in the planning and execution of actions. As an important region of the somatosensory cortex, the right posterior central gyrus has an information-feedback mechanism composed of motor-sensory afferent and efferent pathways. This mechanism regulates and controls motor cortex signals through the SMA (Fink et al., 2014). Therefore, we speculated that the increase of ALFF in the sensorimotor cortex of PD patients reflected its compensation for motor dysfunction. Studies have found increased functional connectivity between the sensorimotor cortex and subthalamic nucleus and the spatial attention network (Kurani et al., 2015; Onu et al., 2015), which validates our view further. We found that, compared with the diffuse malignant subtype, the mild subtype had more extensive compensation in the temporal lobe and involved bilateral brain regions. We also observed a significant negative correlation between ALFF in the right temporal lobe and the HAMD score. Hence, enhancement of spontaneous neuronal activity in the temporal lobe may be a compensatory effect on non-motor symptoms such as depression. Studies have demonstrated that the temporal lobe is associated with depressive symptoms(Ma et al., 2012). Taken together with the studies mentioned above, we believe that patients with the mild subtype may have a more muscular compensatory function. This hypothesis also explains why patients with the mild subtype show relatively mild impairments in motor function and cognitive function.

In the present study, patients with PD of both subtypes had reduced GMV of bilateral vision-related cortices compared with HCs. The bilateral calcarine cortex, lingual gyrus, and cuneus are involved in color recognition, shape recognition, visual memory, and primary processing of visual information. The fusiform gyrus is part of the ventral visual pathway involved in facial recognition. Goldman and coworkers (Goldman et al., 2014) found that damage to the lingual gyrus, cuneus, and fusiform gyrus caused hallucinations. Those observations are consistent with our findings, suggesting that the decrease in GMV in this region may cause visual impairment and hallucinations in PD patients. We also found that GMV decreased in the bilateral thalamus, left hippocampus, and amygdala in PD patients with both subtypes. Studies in PD patients with mild cognitive impairment and dementia have shown a correlation between reduced GMV in this region and cognitive impairment (Bouchard et al., 2008; Fu et al., 2020). In the present study, scores for MoCA and MMSE for the two subtypes in PD patients were lower than those observed in HCs, thereby showing varying degrees of cognitive impairment. Those findings further support the notion that the reduction of GMV in the thalamus, hippocampus, and amygdala are a cause of cognitive impairment in PD patients.

Interestingly, we also found that GMV of the left thalamus in both subtypes was negatively correlated with the UPDRSIII score. Similar findings were documented by Xu and colleagues (Xu et al., 2020), who showed that left-thalamus injury had a significant impact on dyskinesia in PD patients. Compared with HCs, GMV of the bilateral frontal lobe, parahippocampal gyrus, and temporal lobe was decreased in patients with the diffuse malignant subtype, whereas only bilateral inferior temporal gyrus and right middle temporal gyrus were involved in patients with the mild subtype. A meta-analysis showed reduced frontal-limbic system-temporal GMV to be the major feature of cognitive decline in PD patients. The progression of reduced GMV from a unilateral brain region to bilateral brain regions is an important marker of gradual worsening of PD cognitive impairment and progression to dementia (Xu et al., 2016). Our findings are consistent with the conclusions stated above.

Under an identical disease duration, patients with the diffuse malignant subtype had more extensive brain areas with reduced GMV and more severe cognitive impairment than those with the mild subtype. In addition, patients with the diffuse malignant subtype had a negative correlation between GMV and the HAMD score in the left temporal lobe, which suggested that the decrease in GMV in the temporal lobe may be related to the depressive symptoms observed in PD cases. Studies have demonstrated that patients with temporal-lobe epilepsy have a higher likelihood of depression compared with patients with other types of epilepsy (Valente and Busatto Filho, 2013). We also found that both subtypes had reduced GMV in the cerebellum, and studies have shown that the reduction in cerebellar GMV of PD patients involves several functional disorders (e.g., motor, cognitive, and emotional regulation) (Ma et al., 2018). However, we found that the reduced GMV in the left cerebellum of patients with the mild subtype was more severe than that of patients with the diffuse malignant subtype, which differed significantly from the changing trend of GMV in the brain of PD cases with both subtypes. This phenomenon may be because: (i) of the different pathophysiological mechanisms involved in the two subtypes; (ii) the cerebellar damage in PD patients with the mild subtype is more severe than that in PD cases with the diffuse malignant subtype. However, the structural and functional changes involved in the cerebellum in PD are extremely complex, and more targeted studies are needed to identify them.

Our results for clustering analysis using an independent PD dataset are in good agreement with those obtained by Fereshtehnejad and colleagues (Fereshtehnejad et al., 2017) and Belvisi and coworkers (Belvisi et al., 2021). Among them, the diffuse malignant subtype showed more severe impairment in motor function, cognitive function, and brain atrophy than other subtypes. Our study had two main strengths. First, it provides detailed information on brain regions with gray-matter atrophy and spontaneous neuronal activity between different subtypes. Second, use of brain imaging data rather than clinical scales as clustering features reduces the interference of human subjectivity.

Our study had two main some limitations. First, our study cohort was small and all PD subtypes could not be covered. Second, the cumulative effect of long-term anti-PD drugs may have influenced our results.

## Conclusions

Cluster analysis based on multimodal MRI indicators allowed us to identify two PD subtypes. These two PD subtypes showed different neurodegenerative patterns upon imaging. Our results provide a new direction for exploring PD subtypes. Study of the pathophysiological mechanism may provide important clues for the prognosis. Longitudinal studies are needed to examine the stability of our results.

## Data availability statement

The raw data supporting the conclusions of this article will be made available by the authors, without undue reservation.

## Ethics statement

The studies involving human participants were reviewed and approved by Institutional Review Board of China Medical University. The patients/participants provided their written informed consent to participate in this study.

## Author contributions

KC and GF conceived the study, participated in its design, and wrote the manuscript. HP and HY revised important intellectual content. YLi, MG, and YLiu performed the acquisition of data. All authors read and approved the final manuscript.

## Funding

This work was supported by grants from the National Science Foundation of China (82071909).

## Conflict of interest

The authors declare that the research was conducted in the absence of any commercial or financial relationships that could be construed as a potential conflict of interest.

## Publisher's note

All claims expressed in this article are solely those of the authors and do not necessarily represent those of their affiliated organizations, or those of the publisher, the editors and the reviewers. Any product that may be evaluated in this article, or claim that may be made by its manufacturer, is not guaranteed or endorsed by the publisher.
