# Supplementary figures and images for: Identifying and validating subtypes of Parkinson's disease based on multimodal MRI data via hierarchical clustering analysis
Source: Front Hum Neurosci. 2022 Jul 29;16:919081. doi: 10.3389/fnhum.2022.919081 (PMC9372337; doi:10.3389/fnhum.2022.919081)

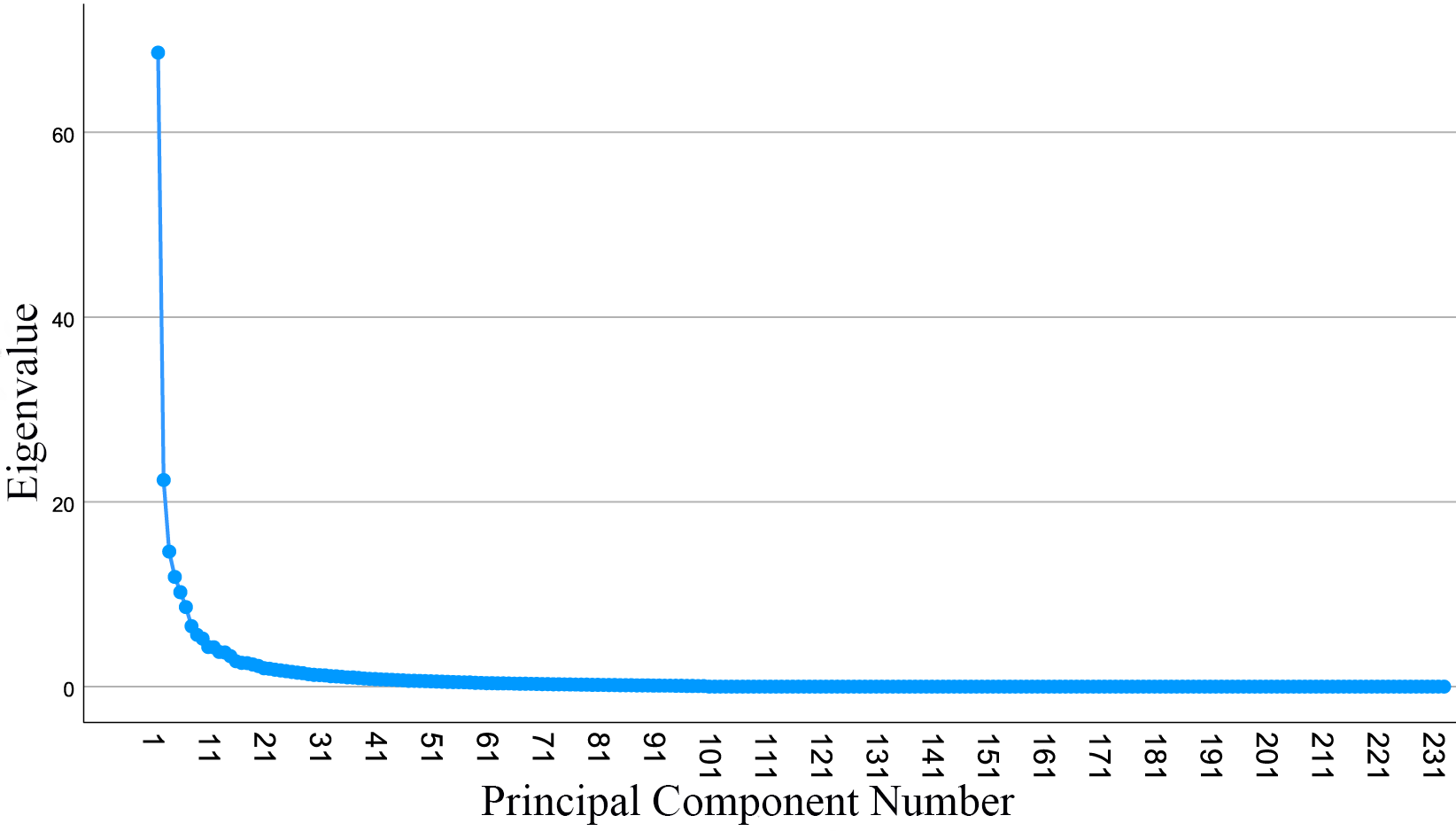

Supplement: Supplementary file 1 [file Image_1.tif]
