# Supplementary material for: Identifying and validating subtypes of Parkinson's disease based on multimodal MRI data via hierarchical clustering analysis
Source: Front Hum Neurosci. 2022 Jul 29;16:919081. doi: 10.3389/fnhum.2022.919081 (PMC9372337; doi:10.3389/fnhum.2022.919081)
Supplement: Supplementary file 2 [file Table_1.docx]

**Supplementary Table 1. Total variance interpretation of PCA dimensionality reduction**

| **Principal Component Number** | **Eigenvalue** | **Percentage of Variance (%)** | **Cumulative(%)** |
| --- | --- | --- | --- |
| 1 | 68.62623875 | 29.58027532 | 29.58027532 |
| 2 | 22.36186856 | 9.638736449 | 39.21901177 |
| 3 | 14.61307557 | 6.298739471 | 45.51775124 |
| 4 | 11.86862611 | 5.115787118 | 50.63353836 |
| 5 | 10.22536781 | 4.407486123 | 55.04102448 |
| 6 | 8.606986455 | 3.709907955 | 58.75093244 |
| 7 | 6.542496242 | 2.820041483 | 61.57097392 |
| 8 | 5.605887011 | 2.416330608 | 63.98730453 |
| 9 | 5.198541978 | 2.240750853 | 66.22805538 |
| 10 | 4.297865922 | 1.852528415 | 68.0805838 |
| 11 | 4.26082402 | 1.836562078 | 69.91714588 |
| 12 | 3.741609472 | 1.612762704 | 71.52990858 |
| 13 | 3.695476831 | 1.592877945 | 73.12278652 |
| 14 | 3.289383056 | 1.417837524 | 74.54062405 |
| 15 | 2.739668303 | 1.18089151 | 75.72151556 |
| 16 | 2.577573691 | 1.111023142 | 76.8325387 |
| 17 | 2.539747248 | 1.094718641 | 77.92725734 |
| 18 | 2.394371594 | 1.032056721 | 78.95931406 |
| 19 | 2.237487249 | 0.964434159 | 79.92374822 |
| 20 | 1.993140849 | 0.859112435 | 80.78286066 |
| 21 | 1.930654945 | 0.832178856 | 81.61503951 |
| 22 | 1.836418635 | 0.791559756 | 82.40659927 |
| 23 | 1.745834779 | 0.752514991 | 83.15911426 |
| 24 | 1.672605203 | 0.720950518 | 83.88006478 |
| 25 | 1.596567013 | 0.688175436 | 84.56824021 |
| 26 | 1.513079893 | 0.652189609 | 85.22042982 |
| 27 | 1.454030295 | 0.626737196 | 85.84716702 |
| 28 | 1.339903624 | 0.577544666 | 86.42471169 |
| 29 | 1.273039009 | 0.548723711 | 86.9734354 |
| 30 | 1.241445159 | 0.535105672 | 87.50854107 |
| 31 | 1.211537379 | 0.522214388 | 88.03075546 |
| 32 | 1.127347456 | 0.485925628 | 88.51668108 |
| 33 | 1.105693549 | 0.476592047 | 88.99327313 |
| **34** | **1.061076056** | **0.457360369** | **89.4506335** |
| 35 | 0.998592332 | 0.430427729 | 89.88106123 |
| 36 | 0.992236836 | 0.427688291 | 90.30874952 |
| … | … | … | … |
| 99 | 0.059724106 | 0.025743149 | 100 |
